# Hypoxia-Inducible Factor-1α Protects Against Intervertebral Disc Degeneration Through Antagonizing Mitochondrial Oxidative Stress

**DOI:** 10.1007/s10753-022-01732-y

**Published:** 2022-09-06

**Authors:** Wen Yang, Chunwang Jia, Long Liu, Yu Fu, Yawei Wu, Zhicheng Liu, Ruixuan Yu, Xiaojie Ma, Ao Gong, Fangming Liu, Yanni Xia, Yong Hou, Yuhua Li, Lei Zhang

**Affiliations:** 1 Department of Orthopaedics, Qilu Hospital of Shandong University, 107 Wenhuaxi Road, Shandong 250012 Jinan, People’s Republic of China; 2 Department of Spinal Surgery, Heze Municipal Hospital, Heze, Shandong 274031 People’s Republic of China; 3 Department of Pathology, Qilu Hospital, Cheeloo College of Medicine, Shandong University, Jinan, Shandong 250012 People’s Republic of China; 4 Department of Orthopaedics, The First Affiliated Hospital of Shandong First Medical University & Shandong Provincial Qianfoshan Hospital, Shandong 250014 Jinan, People’s Republic of China; 5 Department of Orthopedics, Caoxian People’s Hospital, Heze, Shandong 274400 People’s Republic of China; 6 The First Clinical Medical School, Shandong University, Jinan, Shandong 250012 People’s Republic of China; 7 Department of Rheumatology and Immunology, Affiliated Hospital of Shandong University of Traditional Chinese Medicine, Jinan, Shandong 250012 People’s Republic of China; 8 Department of Orthopedics, Affiliated Hospital of Shandong University of Traditional Chinese Medicine, Jinan, Shandong 250012 People’s Republic of China; 9 Department of Rehabilitation Medicine, The First Affiliated Hospital of Shandong First Medical University, Jinan, Shandong 250014 People’s Republic of China; 10 Department of Operating Room, Qilu Hospital, Cheeloo College of Medicine, Shandong University, 107 Wenhuaxi Road, Jinan, Shandong 250012 People’s Republic of China; 11 Tissue Engineering Laboratory, Department of Radiology, Shandong First Medical University, Jinan, Shandong 250014 People’s Republic of China; 12 Shandong Key Laboratory of Rheumatic Disease and Translational Medicine, Shandong First Medical University, Jinan, Shandong 250014 People’s Republic of China

**Keywords:** Intervertebral disc degeneration, Hypoxia-inducible factor-1α, Protective role, TNF-α, IVDD model.

## Abstract

Intervertebral disc degeneration (IVDD) demonstrates a gradually increased incidence and has developed into a major health problem worldwide. The nucleus pulposus is characterized by the hypoxic and avascular environment, in which hypoxia-inducible factor-1α (HIF-1α) has an important role through its participation in extracellular matrix synthesis, energy metabolism, cellular adaptation to stresses and genesis. In this study, the effects of HIF-1α on mouse primary nucleus pulposus cells (MNPCs) exposed to TNF-α were observed, the potential mechanism was explored and a rabbit IVDD model was established to verify the protective role of HIF-1α on IVDD. *In vitro* results demonstrated that HIF-1α could attenuate the inflammation, apoptosis and mitochondrial dysfunction induced by TNF-α in MNPCs; promote cellular anabolism; and inhibit cellular catabolism. *In vivo* results demonstrated that after establishment of IVDD model in rabbit, disc height and IVD extracellular matrix were decreased in a time-dependent manner, MRI analysis showed a tendency for decreased T2 values in a time-dependent manner and supplementation of HIF-1α improved histological and imaginative IVDD while downregulation of HIF-1α exacerbated this degeneration. In summary, HIF-1α protected against IVDD, possibly through reducing ROS production in the mitochondria and consequent inhibition of inflammation, metabolism disorders and apoptosis of MNPCs, which provided a potential therapeutic instrument for the treatment of IVDD diseases.

## INTRODUCTION

As the population ages, intervertebral disc degeneration (IVDD) demonstrates a gradually increased incidence and has developed into a major health problem worldwide [1–3]. In addition to age, the risk factors of IVDD also include inflammatory cytokines, mechanical trauma, genetic susceptibility, lifestyle factors, certain metabolic disorders and so on [4–9]. IVDD is the main cause of disability because it often causes chronic low back pain (LBP) [10, 11]. Current treatments of IVDD mainly include pharmacological and surgical interventions, aiming for managing symptoms and minimizing disability. However, both of them are costly, often result in complications and have questionable efficacy [12]. Thus, more and more studies have focused on new therapies for IVDD [13, 14].

The nucleus pulposus is characterized by the hypoxic and avascular environment, in which hypoxia-inducible factor-1α (HIF-1α) has an important role through its participation in extracellular matrix (ECM) synthesis, energy metabolism, cellular adaptation to stresses and genesis [15, 16]. In the late stage of IVDD, the expression of HIF-1α is significantly decreased, and neovascularization increases the oxygen concentration. A study reports that HIF-1α can attenuate the apoptosis of nucleus pulposus-derived stem cells induced by excessive mechanical load [14]. IVDD is characterized by increased levels of pro-inflammatory cytokines such as TNF, IL-1β and IL-17. These cytokines are secreted by the IVD cells and can promote ECM degradation, changes in IVD cell phenotype and chemokine production, which consequently results in the degeneration of IVD tissues [17]. Tumour necrosis factor-α (TNF-α) can exacerbate the inflammatory process and is demonstrated to be a key regulator in the development of IVDD [18]. However, the effects of HIF-1α on mouse primary nucleus pulposus cells (MNPCs) exposed to TNF-α and the potential mechanism are still not investigated. In this study, the effects of HIF-1α on MNPCs exposed to TNF-α were observed and the potential mechanism was explored, and a rabbit IVDD model was established to verify the protective role of HIF-1α on IVDD.

## RESULTS

### HIF-1α Attenuated TNF-α-Induced Inflammation in Primary Mouse Nucleus Pulposus Cells (MNPCs)

**Fig. 1:**
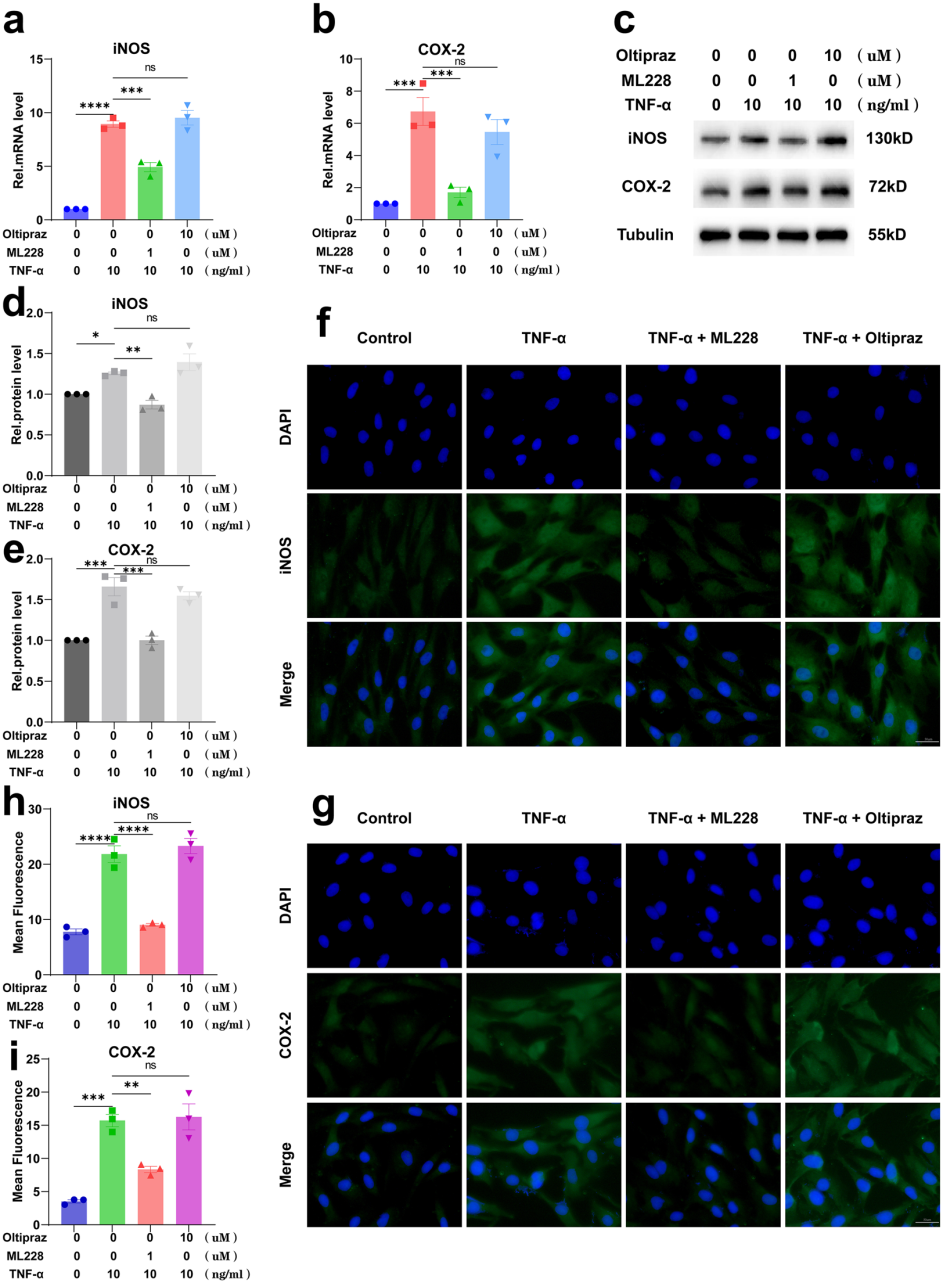
HIF-1α attenuated TNF-α-induced inflammation in primary mouse nucleus pulposus cells (MNPCs). **a**, **b** The mRNA levels of iNOS and COX-2 after cells were treated as above were detected using real-time PCR. **c**–**e** The protein levels of iNOS and COX-2 were detected using Western blotting and **f**–**i** immunofluorescence staining. The scale bar was 50 μm. The values were the mean of at least three independent experiments. *P < 0.05, **P < 0.01, ***P < 0.001 and ****P < 0.0001.

Primary mouse nucleus pulposus cells (MNPCs) were isolated and then co-cultured with TNF-α, TNF-α and ML228 or TNF-α and Oltipraz for 24 h to detect the mRNA levels of COX-2 and iNOS and 48 h to detect their protein levels. As shown in Fig. 1a, b, ML228 (HIF-1α activator) could reduce the elevated mRNA expression levels of iNOS and COX-2 induced by TNF-α while Oltipraz (HIF-1α inhibitor) could not. The Western blotting results (Fig. 1c–e) and immunofluorescence (Fig. 1f–i) were completely consistent with the real-time PCR results. These suggested that HIF-1α significantly alleviated the TNF-α-induced inflammatory response in MNPCs.

### HIF-1α Promoted Cellular Anabolism and Inhibited Catabolism of MNPCs

**Fig. 2:**
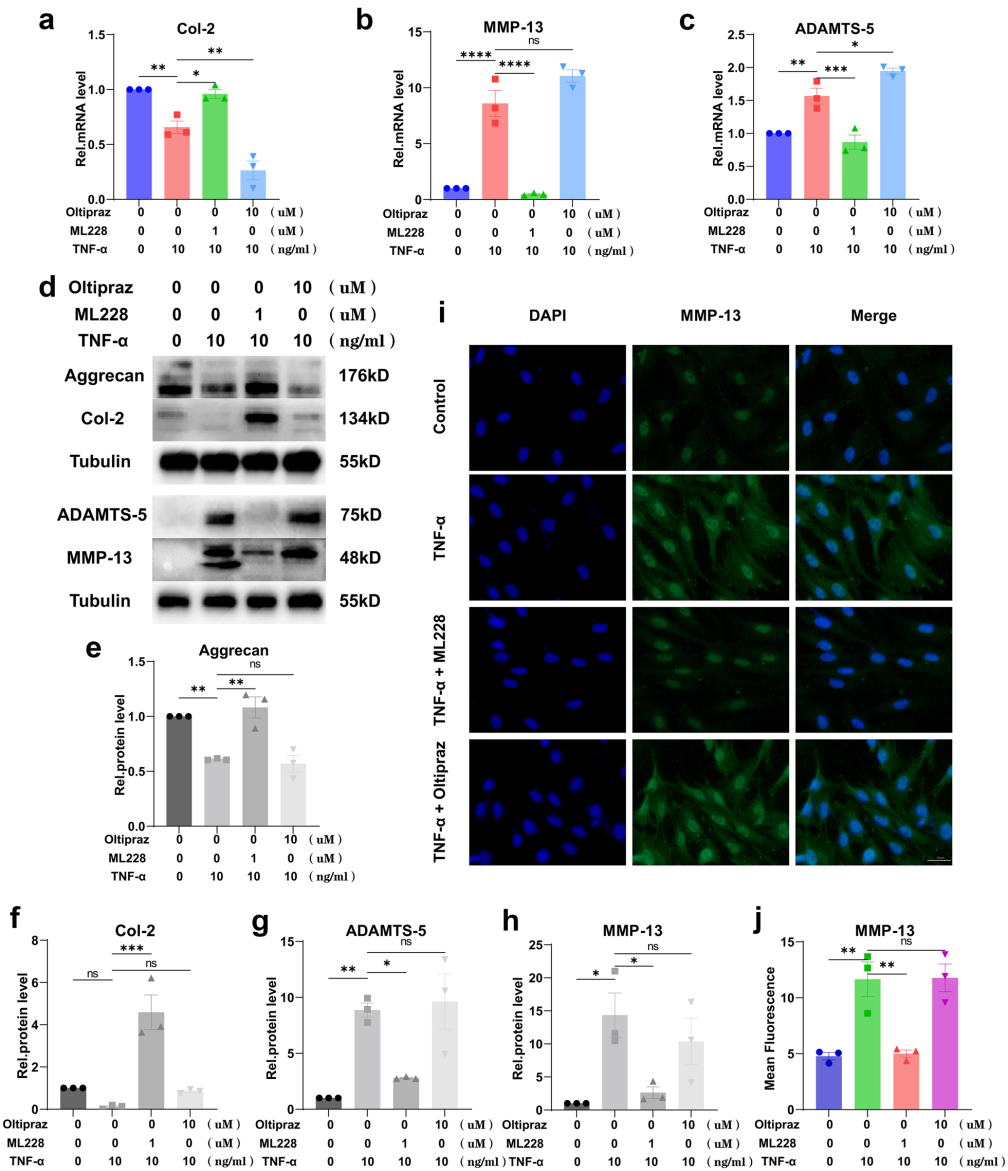
HIF-1α promoted cellular anabolism and inhibited the catabolism of MNPCs. **a**–**c** The expression levels of Col-2, MMP-13 and ADAMTS-5 were detected using real-time PCR. **d**–**h** The protein levels of Aggrecan, Col-2, MMP-13 and ADAMTS-5 were assayed using Western blotting. **i**, **j** The expression levels of MMP-13 were detected by immunofluorescence staining. The scale bar was 50 μm. The values were the mean of at least three independent experiments. *P < 0.05, **P < 0.01, ***P < 0.001 and ****P < 0.0001

Figure 2a–c shows that activation of HIF-1α could upregulate the decreased mRNA levels of Col-2 induced by TNF-α and simultaneously downregulate the increased mRNA levels of MMP-13 and ADAMTS-5 induced by TNF-α while inhibition of HIF-1α could not. Figure 2d–h shows that activation of HIF-1α could upregulate the decreased protein levels of Col-2 and Aggrecan induced by TNF-α and simultaneously downregulate the increased protein levels of MMP-13 and ADAMTS-5 induced by TNF-α while inhibition of HIF-1α could not. Immunofluorescence (Fig. 2i, j) was also performed to demonstrate the expression level of MMP-13, and the results were the same as the above.

### HIF-1α Alleviated the TNF-α-Mediated Apoptosis in MNPCs

**Fig. 3:**
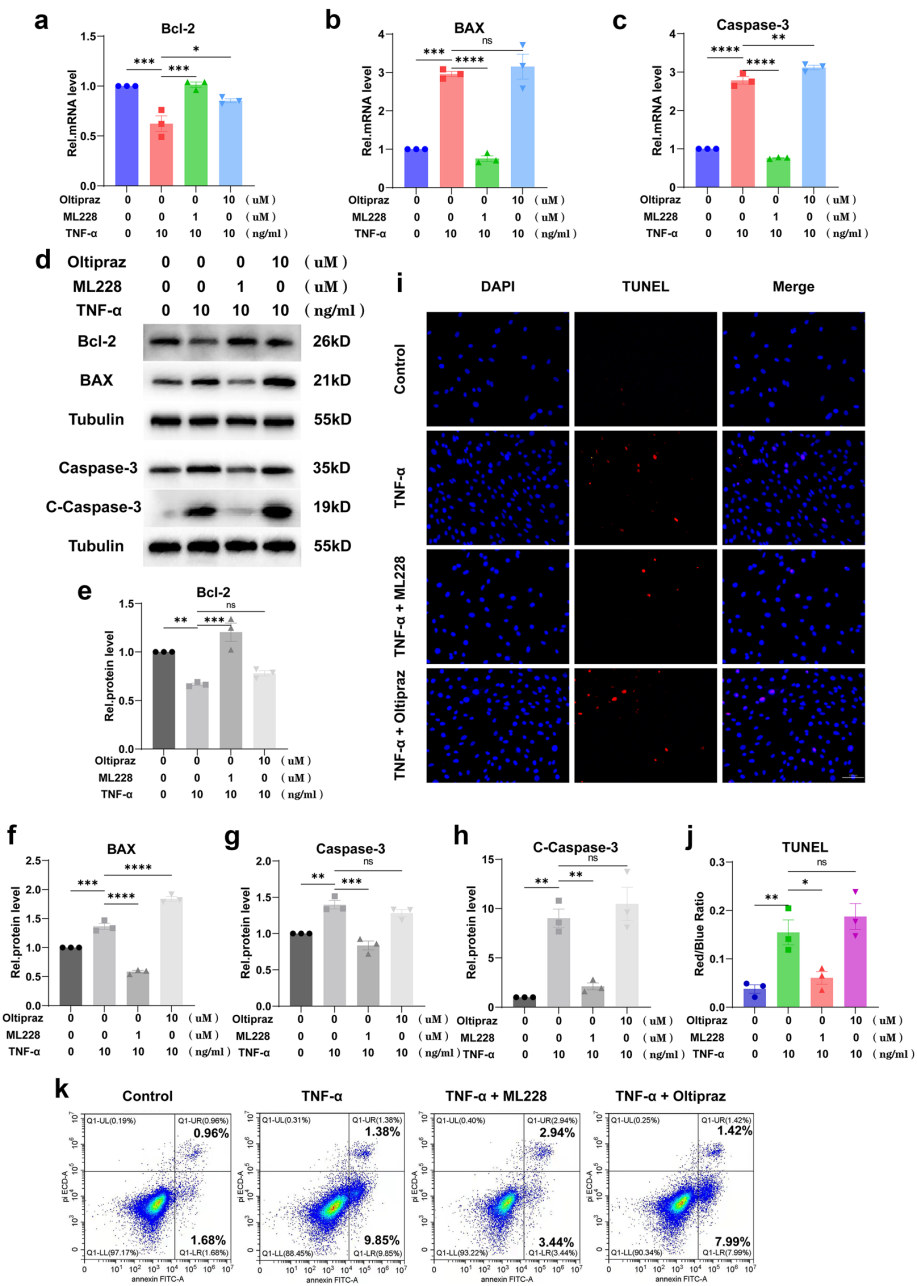
HIF-1α alleviated the TNF-α-mediated apoptosis in MNPCs. **a**–**c** The expression levels of Bcl-2, BAX and Caspase-3 were detected using real-time PCR. **d**–**h** The protein levels of Bcl-2, BAX and C-Caspase-3 were assayed using Western blotting. **i**, **j** TUNEL staining was performed to examine the apoptosis. **k** Flow cytometry was performed to detect apoptosis. The scale bar was 100 μm. The values were the mean of at least three independent experiments. *P < 0.05, **P < 0.01, ***P < 0.001 and ****P < 0.0001.

Figure 3a–c shows that activation of HIF-1α could downregulate the increased mRNA levels of Bax and Caspase-3 induced by TNF-α but upregulate the mRNA level of Bcl-2, while inhibition of HIF-1α could not. Figure 3d–h shows the same results as the above at protein levels by Western blotting, and the inhibitory effect of HIF-1α on cleaved Caspase-3 was particularly significant. Moreover, TUNEL staining of cells (Fig. 3i, j) was performed, which showed that TNF-α promoted cell death while HIF-1α repressed this disorganization. Additionally, flow cytometry (Fig. 3k) was performed to test Annexin/PI to reflect the apoptosis rate of MNPCs, which indicated that TNF-α exaggerated the apoptosis of MNPCs, and this phenomenon could be diminished by HIF-1α.

### HIF-1α Alleviated TNF-α-Induced Mitochondrial Dysfunction in MNPCs

**Fig. 4:**
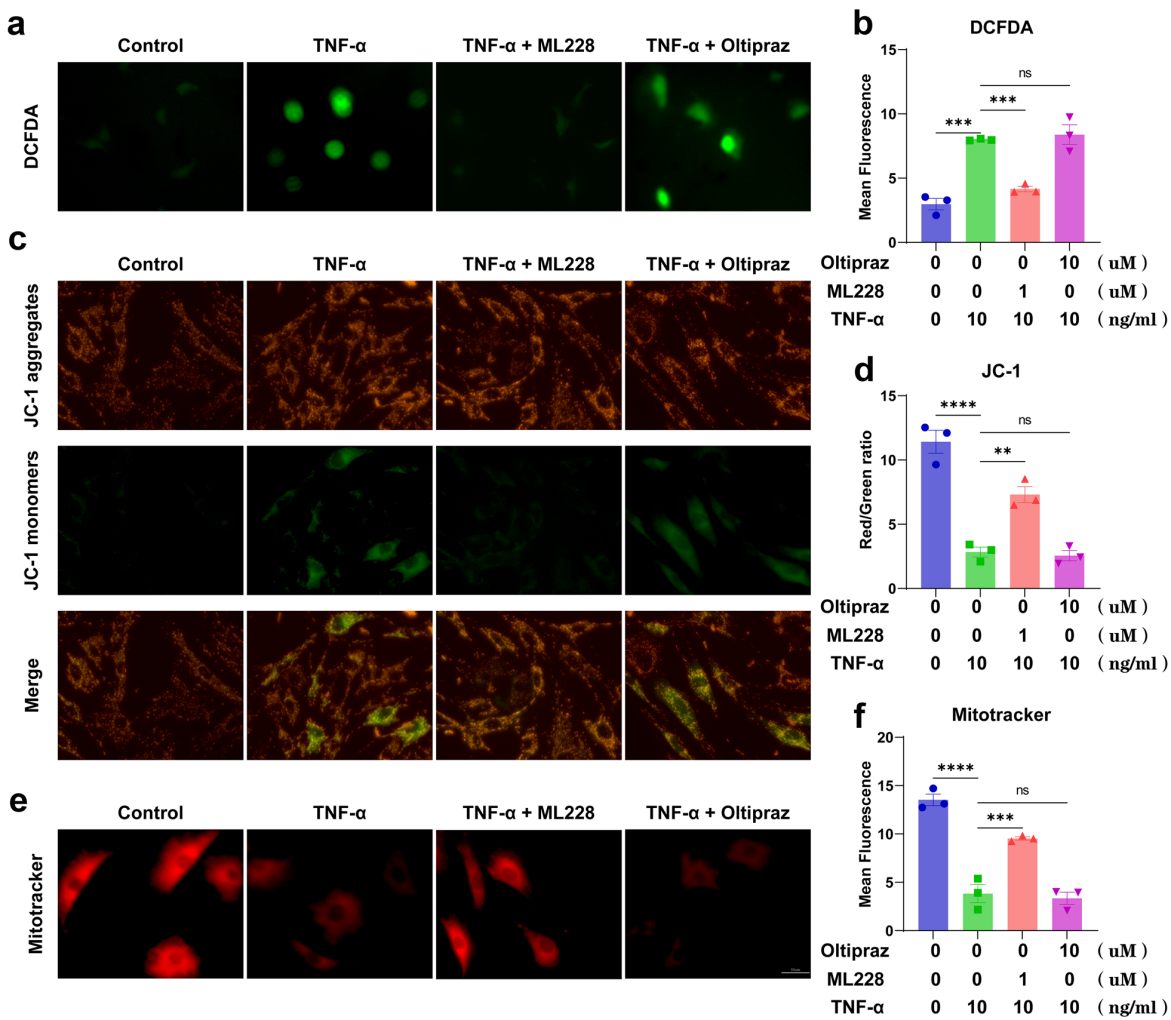
HIF-1α alleviated TNF-α-induced mitochondrial dysfunction in MNPCs. **a**, **b** ROS levels of MNPCs were detected with DCFDA. **c**, **d** JC-1 and **e**, **f** MitoTracker were performed to detect the mitochondrial membrane potential of MNPCs. The scale bar was 50 μm. The values were the mean of at least three independent experiments. *P < 0.05, **P < 0.01, ***P < 0.001 and ****P < 0.0001.

DCFDA assays were used to evaluate ROS synthesis in the mitochondria. As shown in Fig. 4a, b, activation of HIF-1α could decrease the elevated ROS synthesis induced by TNF-α while inhibition of HIF-1α could not. Moreover, JC-1 and Mitotracker assays were performed to detect the membrane potential of the mitochondria (Fig. 4c–f), which showed TNF-α exacerbated the dysfunction of the mitochondria in MNPCs, and activation of HIF-1α could alleviate the mitochondrial dysfunction while inhibition of HIF-1α could not.

### Exogenous Supplementation of HIF-1α Exhibited a Protective Effect on Degeneration of NP Tissue *In Vivo*

**Fig. 5:**
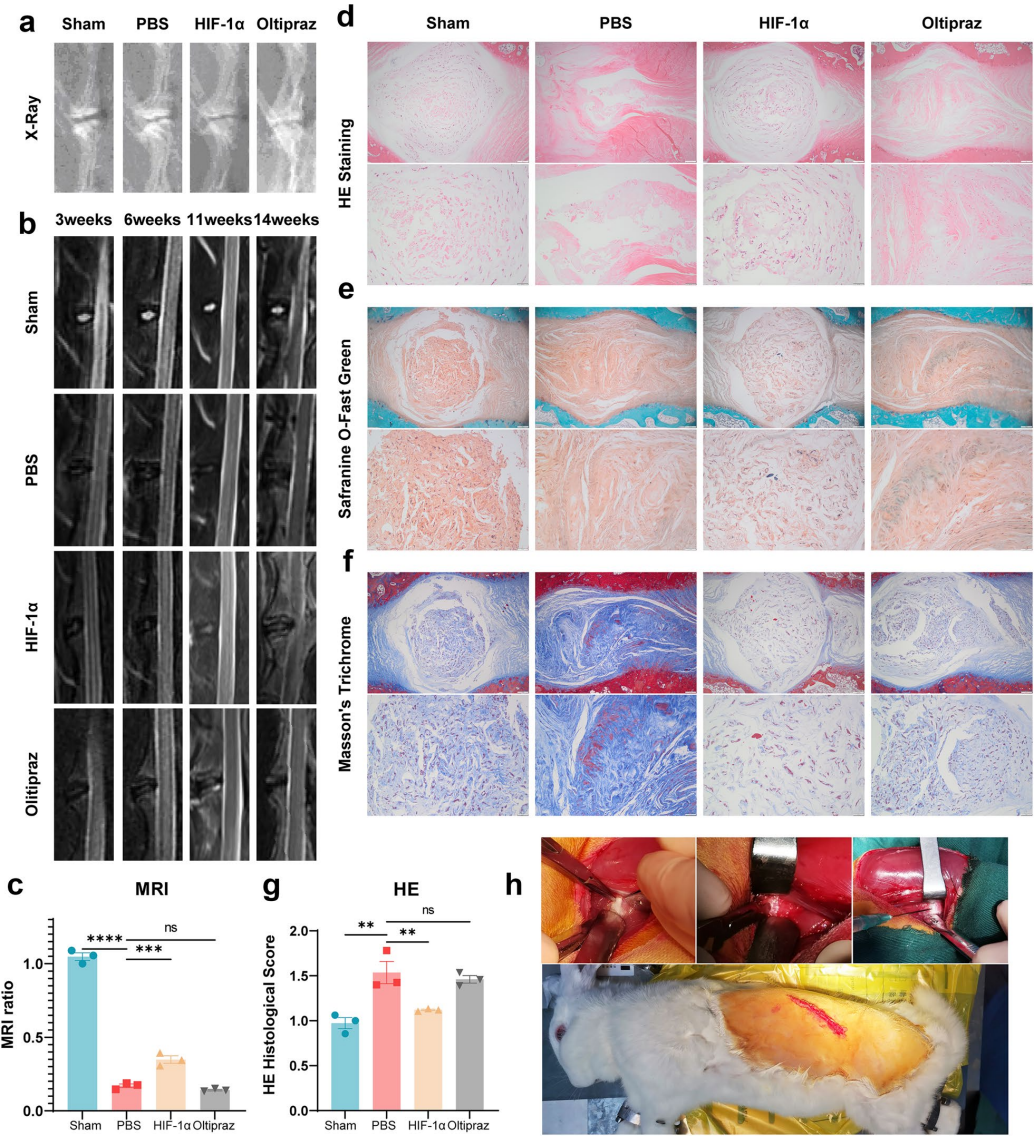
Exogenous supplementation of HIF-1α exhibited a protective effect on the regeneration of NP tissue *in vivo*. **a** X-ray and **b**, **c** MRI to assess the degree of IVDD in rabbits from the sham group, PBS group, HIF-1α group and Oltipraz group. **d**–**g** HE, Safranin O and Masson staining indicated morphological degeneration score such as the height of IVD in this IVDD model was alleviated by supplementation of HIF-1α in comparison with the PBS group and Oltipraz group. Safranin O and Masson staining showed that HIF-1α reduced proteoglycan and collagen loss during the process of IVDD, but suppression of HIF-1α expression aggravated that. The histological scores of each indicated group were calculated according to the grading scale previously published. **h** Photos of the surgical procedure *in vivo*. The scale bar is 500 or 100 μm. The values are the mean of at least three independent experiments. *P < 0.05, **P < 0.01, ***P < 0.001 and ****P < 0.0001.

**Fig. 6:**
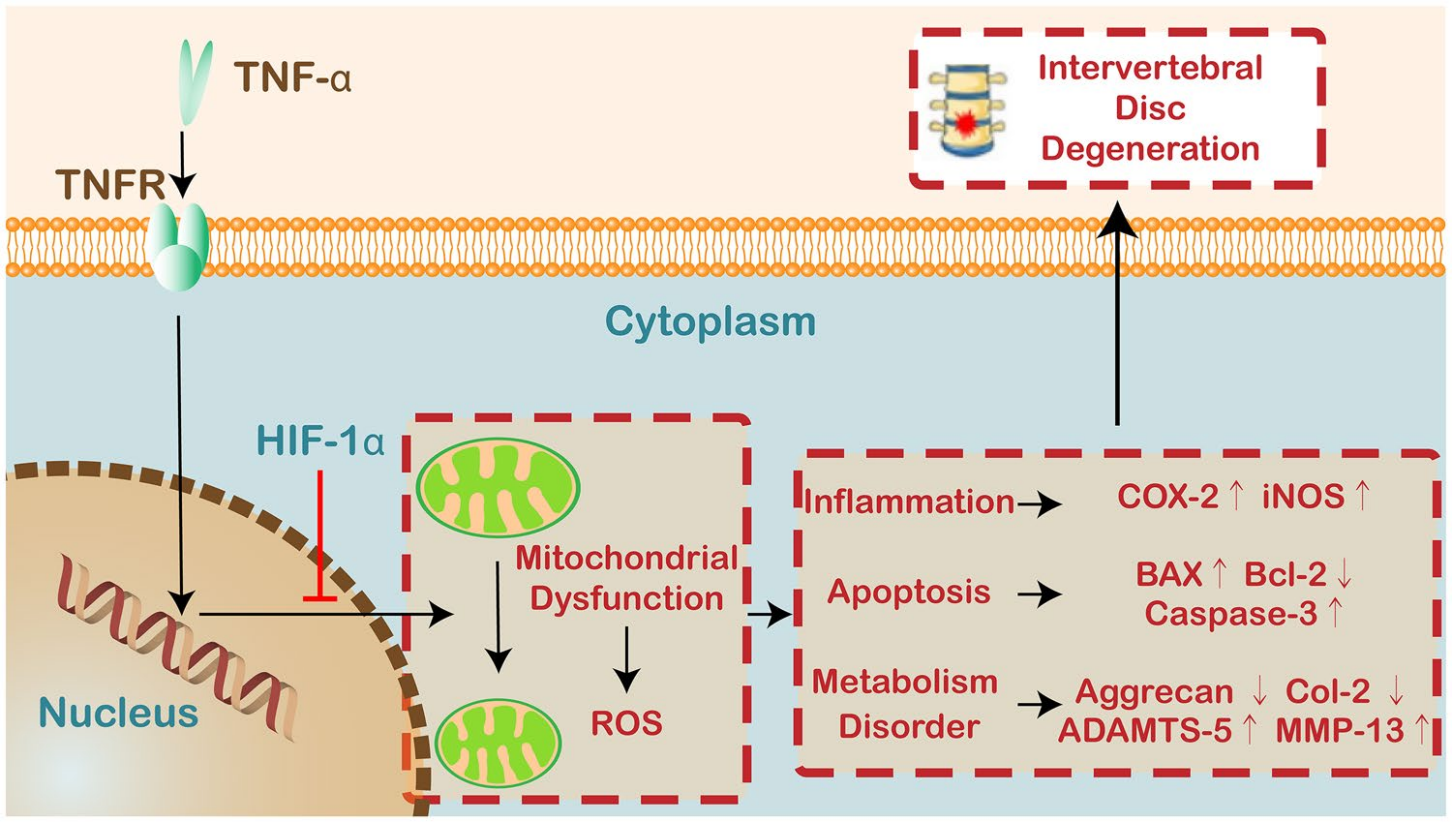
Schematic depicting the proposed model for the role of HIF-1α in IVDD based on this study.

The rabbit IVDD model was established, and HIF-1α recombinant protein or Oltipraz were locally delivered into the NP tissue. Before the rabbits were executed, an X-ray was performed and the IVD height was demonstrated to be reduced, which could be improved by the application of HIF-1α (Fig. 5a). MRI was performed at 3, 6, 11 and 14 weeks, which showed a higher signal intensity of IVD in the HIF-1α supplementation group compared with the PBS group and Oltipraz group (Fig. 5b), and relative signal intensity at week 14 is shown in Fig. 5c. These results suggested that HIF-1α could alleviate the degeneration phenotype of IVD. The IVD samples were then collected for histological analysis. As shown in Fig. 5d–g, HE, Safranin O and Masson staining results indicated that morphological degeneration score such as the height of IVD in this IVDD model was alleviated by supplementation of HIF-1α in comparison with the PBS group and Oltipraz group. Besides, Safranin O and Masson staining results showed that HIF-1α reduced proteoglycan and collagen loss during the process of IVDD while suppression of HIF-1α expression aggravated that (Fig. 6).

## MATERIALS AND METHODS

### Isolation and Culture of Mouse Primary Nucleus Pulposus Cells (MNPCs)

In this study, mice were sacrificed by cervical vertebra dislocation and then soaked in 75% ethyl alcohol for 10 min to disinfect the entire body. After the dorsal hair had been shaved, the whole spine was separated from the back. The disc tissue was separated under a microscope, cut into pieces and placed in culture dishes. The cells were digested with 0.2% collagenase type II (Gibco, USA) at 37 °C for 8 h. The cells were then cultured in DMEM/F12 (HyClone, USA) supplemented with 10% foetal bovine serum (FBS, Gibco, USA), 1% penicillin and streptomycin (P1400, SolarBio, China) under standard incubation conditions (37 °C, 5% CO_2_). The culture medium was replaced every 3 days, and the cells were passaged when they reached 80–90% confluence. The cells from within five generations were used in all vitro experiments. In subsequent experiments, the control-group and TNF-α (HY-P7058, MCE, USA) (10 ng/ml) group were cultured under standard incubation conditions (37 °C, 5% CO_2_), while the ML228 (HY-12754, MCE, USA) (1 μM) group and Oltipraz (HY-12519, MCE, USA) (10 μM) group were cultured under hypoxic conditions (37 °C, 1% O2, 5% CO_2_ and 94% N_2_).

### Western Blotting Analysis

Total protein was extracted from MNPCs of each group with the precooled RIPA Lysis Buffer (P0013C, Beyotime Biotechnology) containing 1 mM PMSF on ice for 30 min. The collected liquid was centrifuged at 12,000 rpm for 15 min at 4 °C, and the supernatant was retained. Protein concentration was detected with a BCA protein assay kit (PC0020, SolarBio). Then, to destroy the 3-dimensional protein structure, the proteins in the loading buffer were heated at 100 °C for 10 min. An equal amount of protein from each sample was separated by SDS-PAGE on 8%, 10% or 12% SDS–polyacrylamide gels and then transferred to a polyvinylidene difluoride (PVDF) membrane (Millipore, USA). After being blocked with QuickBlockTM Blocking Buffer (P0252, Beyotime Biotechnology) for 20 min at room temperature, the membranes were incubated with anti-iNOS(1:1000, 18,985–1-AP, Proteintech), anti-COX-2(1:1000, 27,308–1-AP, Proteintech), anti-Tubulin (1:1000, 10,068–1-AP, Proteintech), anti-Aggrecan (1:1000, 13,880–1-AP, Proteintech), anti-Col-2 (1:1000, 28,459–1-AP, Proteintech), anti-ADAMTs-5 (1:1000, ab41037, Abcam), anti-MMP-13 (1:1000, 18,165–1-AP, Proteintech), anti-Bcl-2 (1:1000, ab196495, Abcam), anti-Bax (1:1000, BM3964, Boster) and anti-Caspase-3 (1:1000, 19,677–1-AP, Proteintech) antibodies at 4 °C overnight. The next day, after washing with Tris-buffered saline Tween-20 (TBST), these membranes were incubated with goat anti-rabbit IgG-HRP secondary antibody (1:5000, Jackson ImmunoResearch) at room temperature for 1 h. Bound antibody was visualized using an enhanced chemiluminescence system (Amersham Life Science, Arlington Heights, IL, USA), and the density of protein bands was quantified using the ImageJ software.

### Real-Time PCR

An RNAfast200 Kit (220011, Fastagen) was used to extract the total RNA from the MNPCs of each group according to the recommended procedure. Total RNA (1 µg) was reverse-transcribed to complementary DNA (cDNA) using HiScript II Q RT SuperMix for qPCR (R222-01, Vazyme). Real-time PCR was carried out with RealStar Fast SYBR qPCR Mix (A301, GenStar). The experiment was repeated three times for each target gene of each group. The nucleotide sequences of the primers are listed in Table 1. The expression levels of target genes were normalized to Tubulin and were calculated by the 2 − ΔΔCT method.

**Table 1:** Primers Used for Quantitative Real-Time PCR

Source	Target	Forward primer, 5′-3′	Reverse primer, 5′-3′
Mouse	COX-2	AATGCTGACTATGGCTACAAAA	AAAACTGATGCGTGAAGTGCTG
	iNOS	ACAGGAGGGGTTAAAGCTGC	TTGTCTCCAAGGGACCAGG
	MMP-13	ACTTTGTTGCCAATTCCAGG	TTTGAGAACACGGGGAAGAC
	ADAMTS-5	GCATTGACGCATCCAAACCC	CGTGGTAGGTCCAGCAAACAGTTAC
	Col-2	ACTAGTCATCCAGCAAACAGCCAGG	TTGGCTTTGGGAAGAGAC
	Bcl-2	TGTGGTCCATCTGACCCTCC	ACATCTCCCTGTTGACGCTCT
	Bax	CTGAGCTGACCTTGGAGC	GACTCCAGCCACAAAGATG
	Caspase-3	AGGAGGGACGAACACGTCT	CAAAGAAGGTTGCCCCAATCT
	β-Tubulin	CAGCGATGAGCACGGCATAGAC	CCAGGTTCCAAGTCCACCAGAATG

### Immunofluorescence Staining

The cells were treated as indicated, and after 24 h, the cells were fixed with 4% paraformaldehyde for 20 min. After being permeabilized with 0.2% Triton X-100 for 20 min, the samples were blocked by BSA at 37 ℃ for 1 h. Then, the cells were incubated with anti-iNOS (1:500, 18985–1-AP, Proteintech), anti-COX-2 (1:500, 27308–1-AP, Proteintech) and MMP-13 (1:500, 18,165–1-AP, Proteintech) antibodies at 4 °C overnight. The next day, the cells were incubated with fluorescently labelled goat anti-rabbit IgG (1:100, Abbkine) for 1 h at 37 ℃. The nuclei were stained with DAPI. The images were taken using a fluorescence microscope (ZEISS Vert. A1) and analysed with the ImageJ software.

### TUNEL Staining

To examine the apoptosis of MNPCs in each experimental group, cells were stained with a TMR (red) Tunel Cell Apoptosis Detection Kit (G1502, Servicebio). All the procedures were performed according to the manufacturer’s instructions. The images were captured using a fluorescence microscope (ZEISS Vert. A1).

### Flow Cytometry

The apoptosis of MNPCs from each group was detected by flow cytometry. Cells were stained with propidium iodide (PI) and Annexin V-FITC for 15 min at room temperature in the dark with a FITC Annexin V Apoptosis Detection Kit (E-CK-A211, Elabscience) according to the manufacturer’s instructions. Cell apoptosis was detected with a CytoFLEX S flow cytometer (Beckman Coulter, USA), and the data obtained were analysed with the CtyExpert software.

### Reactive Oxygen Species Assay

To detect intracellular reactive oxygen species (ROS), we used an ROS assay kit (S0033, Beyotime Biotechnology). All the procedures were performed according to the manufacturer’s instructions. Briefly, after washing twice with sterile PBS, cells were stained with 10 μM DCFDA at 37 °C for 20 min in the dark. Then, the cells were washed with a basal culture medium three times. The images were captured using a fluorescence microscope (ZEISS Vert. A1).

### JC-1 Assay

The mitochondrial membrane potential changes of MNPCs after treatment were detected with a JC-1 assay kit (C2006, Beyotime). Based on the manufacturer’s instructions, each group’s cells were stained with the JC-1 staining solution at 37 °C for 20 min to protect them from light. Then, the cells were washed twice with JC-1 staining buffer, and the images were observed and captured using a fluorescence microscope (ZEISS Vert. A1).

### MitoTracker Assay

MitoTracker staining was performed to visualize the mitochondria and detect the mitochondrial membrane potential of each group following the instructions of the Mito-Tracker Red CMXRos (C1049B, Beyotime Biotechnology). The cells were incubated with the culture medium containing 20 nM Mito-Tracker Red CMXRos for 30 min at 37 ℃ in the dark. Then, the images were captured using a fluorescence microscope (ZEISS Vert. A1) after changing the fresh culture medium.

### X-Ray and Magnetic Resonance Imaging (MRI)

The rabbits in each group were performed an X-ray before execution. Radiographs were captured at a collimator-to-film distance of 66 cm, an exposure of 63 mAs and a penetration power of 35 kv. MRI was performed for each group at 3, 6, 11 and 14 weeks, and T2-weighted images (repetition time: 3000 ms; echo time: 80 ms; field of view: 200 mm^2^; slice thickness: 1.4 mm) were obtained by MRI using a 1.5-T system (GE) in the sagittal plane. The MRI grade of NPs was evaluated as previously reported.

### Histological Staining

The rabbits were sacrificed at 14 weeks after indicated surgery, and the IVD tissues were collected and fixed in 4% paraformaldehyde for 2 days. After decalcification in 10% EDTA (pH 7.2–7.4), the samples were processed, embedded in paraffin and cut into 5-μm sections using a microtome. H&E staining was performed to evaluate the morphological changes of the nucleus pulposus with a H&E Staining Kit (EE0012, Sparkjade), and the histological grading of these samples was evaluated in accordance with the grading scale based on the morphology of AF and the cellularity of NP. Safranin O staining was performed to detect the changes in proteoglycans with a Safranin O staining kit (G1371, SolarBio) according to the manufacturer’s recommended procedure. Masson staining was performed to confirm collagen loss of these samples with a Masson’s Trichrome Stain Kit (G1340, SolarBio) according to the manufacturer’s recommended procedure. The images were captured by a microscope (Leica DMI3000B).

### Surgery Procedure

The protocol of this study was approved by the Institutional Animal Care and Use Committee. Twelve New Zealand white rabbits (female), ranging from 2.5 to 3.0 kg in body weight (Jinfeng Experimental Animal Co. Ltd., Jinan, China), were used in this study. Rabbits were housed in separate cages under standard conditions with a light–dark cycle (12 h-12 h) and dry-bulb room temperature at 22–24 °C and provided ad libitum access to tap water and food pellets daily. Rabbits were anaesthetized by an intravenous injection of 10% chloral hydrate (3 mL/kg). Rabbits were then placed into a left lateral position, and the vertical line for outward 1/3 of the connecting line between the anterior superior spine and navel or the outer margin of the transverse process was chosen as the incision. The external oblique muscle was outwardly separated from the beginning of its fascia to find the outer margin of the longissimus muscle, the deep fascia was cut open to locate the reference transverse process, the transverse abdominis was separated to expose the psoas major, and the psoas major was retracted toward the abdomen and the position was leaned 20° toward the back to expose the vertebral body. The lumbar spine’s lowest levels (L6–L7) should be avoided to eliminate possible influences of the lumbosacral junction. After the nucleus pulposus was removed, HIF-1α recombinant protein (11977-H07E, Sino Biological) was injected into the target intervertebral disc in the HIF group, and Oltipraz (HY-12519, MCE, USA) was injected into the target intervertebral disc in the HIF inhibitor group. PBS was injected into the target intervertebral disc in the PBS group. The sham group only underwent surgery, but no substance was injected into the intervertebral disc. MRI examinations were performed at 3, 6, 11 and 14 weeks postoperatively, and X-ray examinations were performed before execution. After euthanasia, disc specimens were obtained, and histological analysis was performed.

### Statistical Analysis

Analysis of data was performed with GraphPad Prism (GraphPad Software Inc., USA). Comparisons of various groups were performed using analysis of variance (ANOVA) with Tukey’s post hoc test. Data were presented as “mean ± SEM”. Statistical significance was indicated when two-sided *P* < 0.05.

## DISCUSSION

*In vitro* results demonstrated that HIF-1α could attenuate the inflammation, apoptosis and mitochondrial dysfunction induced by TNF-α in MNPCs, promote cellular anabolism and inhibit cellular catabolism.

TNF-α is an important pro-inflammatory cytokine which can stimulate an inflammatory cascade through binding to the TNFR [17, 19–21]. Its level is significantly elevated in the disc tissue and peripheral serum of patients with IVDD [22–24]. TNF-α has been shown to be associated with a variety of pathological IDD processes such as inflammatory cascade, ROS production, apoptosis, ECM degradation, pyroptosis and proliferation. These indicate their critical role in the development of IVDD [25, 26]. Additionally, it can also induce an inflammatory response in the nucleus pulposus, leading to an increase of iNOS and COX2 and acceleration of intervertebral disc destruction [27]. Therefore, MNPCs stimulated with TNF-α were used to investigate the effects of HIF-1α on protecting against IVDD in this study. Through co-culturing MNPCs with TNF-α, TNF-α and ML228 or TNF-α and Oltipraz, the results showed that activation of HIF-1α could downregulate the expression of iNOS and COX-2 induced by TNF-α while inhibition of HIF-1α could counteract the effect. This suggested that HIF-1α could possibly attenuate the inflammatory response in IVDD.

Degradation of the nucleus pulposus extracellular matrix is an important cause of IVDD [28]. TNF-α can cause IVDD by promoting the expression of MMP-13 and ADAMTS-5 to enhance catabolism and inhibiting the synthesis of Aggrecan and Col-2 to reduce anabolism. Studies have found that HIF-1α is closely related to the synthesis of ECM in chondrocytes [29, 30]. In our study, activation of HIF-1α could inhibit catabolism through downregulating the elevated expression of MMP-13 and ADAMTS-5 induced by TNF-α and enhance anabolism through upregulating the decreased expression of Aggrecan and Col-2 induced by TNF-α while inhibition of HIF-1α could not.

Cellular senescence and death occur widely in various tissues, including apoptosis, necrosis and autophagy, and the loss of nucleus pulposus cells due to apoptosis is one of the important causes of IVDD [31]. Studies have reported that massive death of NP cells and significant degeneration of IVD were observed in mice after conditional knockout of the HIF-1α gene [32, 33]. It is well known that TNF-α can induce the apoptosis of NP cells through increasing the expression of proapoptotic cytokines such as BAX and C-Caspase-3 and reducing the expression of antiapoptotic cytokines such as Bcl-2 [34–36], which has been considered as a potential target for investigation of IVDD. In our study, activation of HIF-1α could downregulate the increased expressions of BAX and C-Caspase-3 induced by TNF-α and upregulate the decreased expression of Bcl-2 induced by TNF-α while inhibition of HIF-1α could counteract the effect. Moreover, TUNEL and flow cytometry also showed the attenuated effect of HIF-1α on the apoptosis of NP cells induced by TNF-α.

The mitochondria, as the centre of cellular energy metabolism, is involved in a variety of signalling pathways and regulates cellular function and survival [37]. After the mitochondria is exposed to adverse stimulation, multiple harmful changes will occur such as increased oxidative stress, swelling and deformation and decreased membrane potential [38, 39]. These changes can lead to inflammasome activation, cell senescence and death, which plays a crucial role in some degenerative diseases [40]. Studies have reported abnormal mitochondrial morphology and dysfunction in ageing NP cells, and mitochondrial dysfunction plays a detrimental role in the development of IVDD [41]. TNF-α can cause damage to the mitochondrial structure, such as swelling and deformation in NP cells [42]. HIF-1α is a transcription factor that responds to the reduction of intracellular oxygen concentration and can enhance cellular resistance to oxidative stress as an endogenous anti-oxidative stress regulator [43]. In our study, activation of HIF-1α reversed the enhanced ROS production and impaired membrane potential in the mitochondria induced by TNF-α while inhibition of HIF-1α was not.

In addition to *in vitro* experiments, we also evaluated the radiological and histological changes in rabbit IVDs through modulation of local HIF-1α activity in IVD for the first time. After the establishment of the IVDD model in rabbits, disc height and IVD extracellular matrix were decreased in a time-dependent manner, and MRI analysis showed a tendency for decreased T2 values in a time-dependent manner, which is in line with previous findings. Intriguingly, our results indicated that supplementation of HIF-1α improved histological and imaginative IVDD while downregulation of HIF-1α exacerbated. These results further confirmed the conclusions of *in vitro* experiments.

In summary, HIF-1α protected against IVDD, possibly through reducing ROS production in the mitochondria and consequent inhibition of inflammation, metabolism disorders and apoptosis of NP cells, which provided a new idea for exploring the therapeutic strategies and targeted drugs for IVDD (Fig. 6).

The limitations of this study mainly included two aspects. The first was a small sample size for model rabbits, and the other was that the potential mechanism associated with the protective role of HIF-1α for IVDD was not verified *in vivo*.

## Data Availability

The datasets generated and/or analysed during the current study are available from the corresponding authors on reasonable request.
